# Adherence to fecal immunochemical test screening among adults at average risk for colorectal cancer

**DOI:** 10.1007/s00384-021-04055-w

**Published:** 2021-11-02

**Authors:** Deborah A. Fisher, Nicole Princic, Lesley-Ann Miller-Wilson, Kathleen Wilson, Kathryn DeYoung, A. Burak Ozbay, Paul Limburg

**Affiliations:** 1 Division of Gastroenterology, Duke University School of Medicine, 3100 Tower Blvd, Durham, NC 27707 USA; 2 IBM Watson Health, 75 Binney St, Cambridge, MA 02142 USA; 3 Exact Sciences Corporation, 441 Charmany Dr, Madison, WI 53719 USA; 4 Division of Gastroenterology and Hepatology, Mayo Clinic, 200 1st St SW, Rochester, MN 55905 USA

**Keywords:** Colorectal neoplasms, Mass screening, Guideline adherence

## Abstract

**Purpose:**

This study examined adherence to screening for fecal immunochemical test (FIT).

**Methods:**

Adults (≥ 50–75) with a FIT between 1/1/2014 and 6/30/2019 in MarketScan administrative claims were selected (index = earliest FIT). Patients were followed for 10 years pre- and 3 years post-index. Patients at increased risk for CRC or with prior screening were excluded. Year over year adherence was measured post-index.

**Results:**

Of 10,253 patients, the proportion adherent to repeat testing at year 2 was 23.4% and 10.6% at year 3. Of 76.6% not adherent in year 2, 5.4% were adherent in year 3.

**Conclusion:**

Results suggest adherence to FIT tests is poor, minimizing potential benefits. Future studies are needed to consider alternative test options and whether more choice will improve long-term adherence.

## Objective

Guideline organizations such as the United States Preventive Services Task Force recommend that colorectal cancer (CRC) screening for average-risk, asymptomatic adults begin at age 50 and continue until age 75 [1]. Although colonoscopy remains the most frequently used screening test for CRC, the fecal immunochemical test (FIT) is a common non-invasive CRC screening option; however, the clinical effectiveness of this method is dependent on long-term adherence to annual testing [2, 3]. The present retrospective, claims-based study examined adherence to FIT over three consecutive cycles of annual screening among a population of screen eligible adults.

## Methods

Average-risk adults, ages 50–75 years, and who had a procedure code for FIT testing between January 1, 2014 and June 30, 2019 were identified in the IBM MarketScan Commercial and Medicare Supplemental Databases. The index date was the date of the first claim for FIT during the study eligibility period. Patients were required to have 10 years of continuous enrollment prior to the index date to access average risk status and to ensure patients were due for screening. To identify average-risk participants, we excluded participants with a history of benign or malignant colorectal neoplasms, inflammatory bowel disease, a known family history of gastrointestinal cancer, or evidence of blood in the stool in the three months preceding the index date. To ensure patients were due for screening, participants were required to have no evidence or FIT or fecal occult blood testing (FOBT) in the year prior to index date, no evidence of multi-target stool DNA test in 3 years prior to index date, no evidence of other screening (i.e., flexible sigmoidoscopy, CT colonography or double contrast barium enema) in 5 years prior to index date, and no evidence of colonoscopy in 10 years prior to index date. Although, this study was designed to assess non-invasive CRC screening modalities, however, due to sample size limitations, the final analysis was restricted to participants screened with FIT. In addition, participants with evidence of any non-FIT CRC screening modality during the 3-year data collection period used to measure adherence were excluded.

Adherence to FIT was examined over three time periods: index (T0), first follow-up screening window (T1), and second follow-up screening window (T2). FIT adherence in T1 was defined as completion of a FIT at months 12–15, allowing for a 3-month grace period for delayed screening ^4^. Adherence in T2 was defined as completion of a FIT at 12–15 months after the T1 screening (among those who completed T1 screening) or at months 16–36 among those who did not complete T1 screening. Adherence patterns were defined as consistently adherent (FIT at both T1 and T2), consistently nonadherent (no FIT at either T1 or T2), and partially adherent (FIT at only T1 or T2). The median months between tests were also reported.

Because this study used only de-identified medical records and did not involve the collection, use, or transmittal of individually identifiable data, it is not considered protected health information under the HIPAA Privacy Rule and is exempt from Institutional Review Board approval.

## Results 

In total, 10,253 participants met the defined study eligibility criteria. The mean participant age was 56.0 years, with sex distributions of 32.8% men. The large majority of participants were commercially insured: 94.7%. Mean (SD) Deyo-Charlson comorbidity score was 0.4 (0.9).

**Fig. 1:**
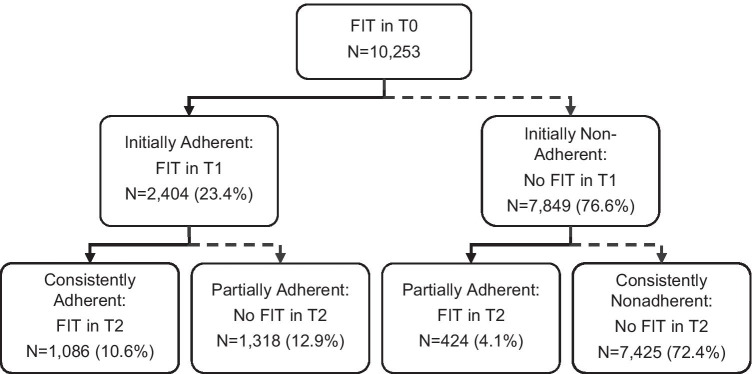
Adherence to screening by fecal immunochemical testing (FIT). Adherence to FIT was defined as consistently adherent (FIT in both T1 and T2), consistently nonadherent (no FIT in either T1 or T2), and partially adherent (FIT in only T1 or T2). In total, 23.4% of participants had a repeat FIT in T1, and only 10.6% of participants completed a repeat FIT in T1 and T2. T0: Index FIT screening. T1: First follow-up screening window of months 12–15 after index FIT screening. T2: Second follow-up screening window of months 12–15 after FIT in T1 or months 16–36 after T0 (if no FIT in T1)

For participants initiating FIT, 23.4% were adherent in T1, while 76.6% were non-adherent in T1 (Fig. 1). Over the full follow-up, 10.6% of participants were consistently adherent, 72.4% were consistently nonadherent, and 17.0% were partially adherent. The median time between tests was 12.7 months.

## Discussion

FIT screening combined with organized patient outreach has been shown to increase overall adherence to CRC screening and contribute to early detection and prevention of CRC [5]. However, reported longitudinal adherence rates vary. Repeat FIT adherence rates of 75.3% to 86.1% have been achieved in an organized screening program; [3] whereas, rates of 15.8% to 28.8% were reported from a safety-net health system [4]. In this study analyzing data from diverse health plans, 23.4% of participants had a repeat FIT in the first follow-up window, and only 10.6% of participants completed a repeat test during both follow-up windows.

This study is subject to the limitations common to all retrospective administrative claims studies, such as lack of independent confirmation that a test was completed. This study excluded those who switched screening modalities during the 3-year data collection period to ensure a measure of adherence the FIT test, however, it does not take into account that patients may be screened (and be adherent) using other test types after an initial FIT test.

Results from this analysis of robust claims data across many health plans suggest that longitudinal adherence to annual FIT is suboptimal for many insured individuals who initially pursue this screening strategy. Data reported from healthcare settings with organized screening programs suggest that considerable infrastructure and outreach is needed to achieve long term adherence within a FIT screening program [6]. Despite this, many modeling studies may assume an impractical, 100% adherence to FIT and other guideline-endorsed screening strategies, which does not reflect real-world, population-level experience. The data from this study can be used to develop models to more accurately estimate the potential benefits achievable with FIT-based screening strategies.

In conclusions, real-world adherence to FIT is suboptimal and this low adherence should be considered when developing models of CRC screening benefits. Future studies are needed to consider alternative CRC screening test options and whether more choice/flexibility will improve long-term adherence.
